# Microbe–Host Interactions are Positively and Negatively Regulated by Galectin–Glycan Interactions

**DOI:** 10.3389/fimmu.2014.00284

**Published:** 2014-06-18

**Authors:** Linda G. Baum, Omai B. Garner, Katrin Schaefer, Benhur Lee

**Affiliations:** ^1^Department of Pathology and Laboratory Medicine, David Geffen School of Medicine at University of California Los Angeles, Los Angeles, CA, USA; ^2^Department of Microbiology, Immunology and Molecular Genetics, David Geffen School of Medicine at University of California Los Angeles, Los Angeles, CA, USA

**Keywords:** galectin, cell surface glycans, microbial pathogen, virus, bacteria

## Abstract

Microbe–host interactions are complex processes that are directly and indirectly regulated by a variety of factors, including microbe presentation of specific molecular signatures on the microbial surface, as well as host cell presentation of receptors that recognize these pathogen signatures. Cell surface glycans are one important class of microbial signatures that are recognized by a variety of host cell lectins. Host cell lectins that recognize microbial glycans include members of the galectin family of lectins that recognize specific glycan ligands on viruses, bacteria, fungi, and parasites. In this review, we will discuss the ways that the interactions of microbial glycans with host cell galectins positively and negatively regulate pathogen attachment, invasion, and survival, as well as regulate host responses that mitigate microbial pathogenesis.

## Introduction

Lectins are proteins that recognize glycan ligands. In mammalian hosts, different types of endogenous lectins can bind to self glycans, e.g., selectins that tether circulating leukocytes to the endothelium or siglecs that regulates signaling in leukocytes by binding to cell surface glycoprotein receptors (1–4). Also in mammalian hosts, various types of lectins can recognize glycans that are typically found on pathogens, but not on host cells, such as mannose binding protein and dectin-1 that bind high mannose and β-glucan ligands, respectively, on yeast (5–8). Thus, these types of lectins have been grouped in the category of pattern recognition receptors (PRRs) that bind pathogen-associated molecular patterns (PAMPs) expressed exclusively on microbes (9, 10).

However, it is becoming increasingly clear that there are several lectins that cannot be exclusively segregated into either category of lectins that only recognize self or lectins that only recognize non-self. These include some C-type lectins, such as DC-SIGN, DCIR, and the macrophage mannose receptor, that can recognize both host and microbial glycans (11–13). Similarly, several members of the galectin family can recognize both host and microbial glycans (14–17). Many C-type lectins and galectins are considered to be components of the innate immune response to pathogens, because binding of these lectins to microbial glycans can either directly or indirectly promote host defense, by triggering leukocyte activation, phagocytosis, complement fixation, and cytokine production (7, 8, 14). However, some of these same lectins can also enhance microbial infection of different hosts, an effect that would seem to be counter-intuitive if lectins are considered to act solely as host defense molecules (7, 17). Thus, the effects of specific lectins as pro-microbe or pro-host must always be considered in a context-dependent manner.

This review will examine roles of galectin family members in both promoting microbial infection and enhancing host resistance to infection. The galectins are a family of lectins with a common carbohydrate recognition domain (CRD), and are found in all multicellular organisms, including fungi, nematodes, insects, and vertebrates. Importantly, all galectins are either bivalent or can multimerize into dimers, pentamers, and higher order oligomers; the multivalency of galectins allows these molecules to bind multiple glycan ligands on either the same cell or on opposing cells, or, in some cases, on microbes and host cells (14, 15, 18).

**Figure 1:**
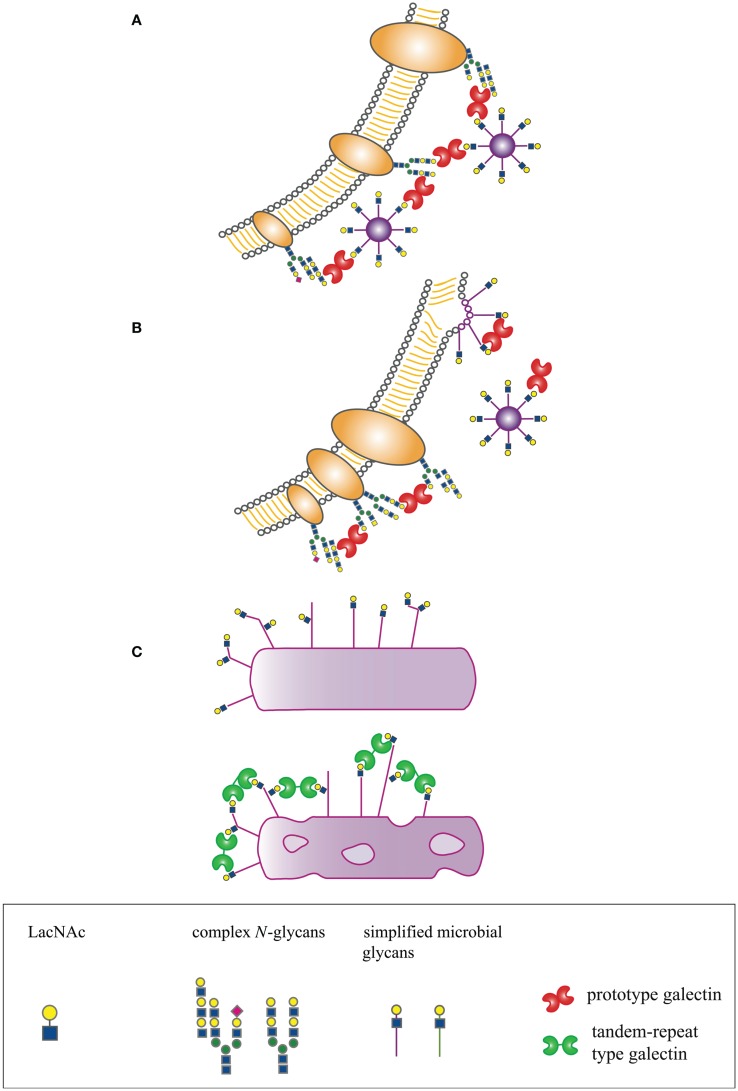
**Host-derived galectins can positively and negatively regulate microbe–host interactions**. **(A)** Galectins can promote microbial attachment to and infection of host cells by bridging pathogen glycans to host cell surface glycans. **(B)** Galectins can inhibit microbial attachment and infection by binding to host or microbial glycans and blocking molecular interactions essential for microbial binding and entry, or by binding to glycans on nascent microbes, such as budding viruses, and preventing proper structural assembly. **(C)** Galectins can be directly microbicidal, an effect regulated by expression of specific glycan ligands on the pathogen surface.

In many organisms, galectins have been described as participating in recognition and defense against microbial pathogens. Several recent reviews have addressed the indirect effects of galectins on microbial pathogenesis through regulation of innate and adaptive immunity, e.g., promoting dendritic cell maturation and migration, enhancing cytokine production, or initiating release of intracellular mediators such as histamine (14, 15, 19–21). In contrast, we will specifically address mechanisms by which galectins directly interact with microbial pathogens to effect three distinct outcomes – enhancement of microbial infection, blockade of microbial infection, or microbicidal activity (Figure 1).

## Galectins Promote Microbial Infection

The best-described role for galectins in microbial infections is to promote pathogen attachment to or entry into host cells. As galectins are typically multivalent, galectins can bind to glycan ligands on both the microbial pathogen and the host plasma membrane to “bridge” the microbe to the target cell. This effect has been described for viruses, bacteria, and parasites.

Sato and colleagues demonstrated that galectin-1 can promote HIV-1 infection of human T cells and macrophages by promoting viral adsorption to the target cells (22, 23). This effect was specific for galectin-1, as galectin-3 did not enhance HIV-1 infection of T cells or macrophages. Moreover, galectin-1 promoted viral attachment, but did not affect fusion, supporting the “bridging” function of galectin-1 in this context. Glycoprotein receptors for galectin-1 have been proposed to be gp120 on the virus and CD4 on the host cells. However, galectin-1 can also bind to CD43 and CD45 on T cells (24, 25); these large glycoproteins are very abundant on the plasma membrane of these cells, and may also contribute to adsorption of virus to target cells. In addition, it is known that budding viral particles can incorporate host cell glycoproteins in the viral envelope, raising the possibility that host cell glycoproteins such as CD45 on the viral envelope may also contribute to galectin-1 binding (26). A similar mechanism of galectin-1 bridging virus to host target cells has recently been observed by our labs for the Nipah virus. Galectin-1 binds to specific glycans on viral envelope glycoproteins and on the target cell plasma membrane to increase adsorption of virus to target cells, resulting in increased efficiency of infection of target cells (Garner et al., in preparation).

Our labs also found that HIV infection of target cells was promoted by another galectin family member, galectin-9 (27). However, in this case, enhanced infection was not achieved by direct “bridging” of the virus to target T cells. Rather, galectin-9 regulated the redox environment at the T cell surface by retaining the thioreductase protein disulfide isomerase (PDI) on the cell surface; as reduced forms of both the viral Env glycoprotein gp120 and the T cell counterreceptor CD4 promote viral entry, the enhancing effect was likely due to the reducing environment created by galectin-9 retention of PDI.

Both galectin-1 and -3 have been implicated in increasing target cell infection with human T cell leukemia virus type 1 (HTLV-1) (28). As described for HIV, galectin-1 was found to stabilize attachment of HTLV-1 to human T cells, resulting in increased efficiency of infection as well as increased fusion of HTLV-1 infected cells. Galectin-3 was also found to increase T cell infection by HTLV-1, although the proposed mechanism differs from the direct bridging of virus to host cell that has been proposed for galectin-1 and HTLV-1. HTLV-1 forms extracellular viral assemblies, and the attachment of these viral assemblies to host T cells is promoted by extracellular matrix proteins such as agrin and collagen, as well as by cell surface proteins including galectin-3. Unlike the HIV envelope glycoprotein, the HTLV-1 env protein is poorly glycosylated, so that galectin-3 may bind to these viral assemblies via recognition of glycans on T cell-derived glycoproteins in these supramolecular aggregates (29). However, in both cases, the galectin-mediated interaction results in increased infection of human T cells with HTLV-1.

A similar bridging function has been proposed for galectin-3 in promoting infection of human corneal epithelial cells by herpes simplex virus (HSV)-1 (30). Galectin-3 specifically bound to HSV-1 in a carbohydrate-dependent manner, but did not bind the highly related HSV-2, indicating that specific glycans on viral envelope glycoproteins are determinants of galectin-3 recognition. Reduction of galectin-3 expression on corneal keratinocytes reduced HSV-1 binding and infection of the cells, indicating that galectin-3 can directly promote HSV-1 infection of target cells.

Direct bridging of pathogen to host target cells has also been described for enhancement of bacterial adhesion to host cells by galectins. Galectin-3 specifically increased binding of *Neisseria meningitidis* to human monocytes and macrophages, but not to nasopharyngeal epithelial cells (31). Galectin-3 bound *N. meningitidis* lipopolysaccharide (LPS) that bears terminal LacNAc sequences, a preferred glycan ligand for galectin-3. Full-length galectin-3 was required for this effect; as proteolytic removal of the N-terminal multimerization domain abrogated the enhanced binding, this implied that galectin-3 must multimerize for enhancement to occur. In addition, galectin-3 null mice demonstrated reduced levels of bacteremia, compared to wildtype mice, after challenge with live *N. meningitidis*, further implicating galectin-3 in promoting bacterial infection of host cells. Similarly, LPS from *Pseudomonas aeruginosa* also bound galectin-3; galectin-3 is produced by human corneal epithelial cells, a target of *P. aeruginosa* infection. Antibodies specific for either the outer core region of *P. aeruginosa* LPS or for galectin-3 blocked binding of bacteria to cultured human corneal epithelial cells, implicating galectin-3 in corneal infection and development of bacterial keratitis (32).

Numerous studies have also found roles for galectins in bridging parasite pathogens to host cells. *Leishmania major* can bind both galectin-3 and -9 (33, 34). Galectin-9 binding to polygalactose epitopes on *L. major* lipophosphoglycans promotes binding of the parasite to macrophages; specific galectin-9 receptors on the target cells were not identified, although the authors proposed that other macrophage lectins, such as the macrophage mannose receptor, may be involved. In contrast to galectin-9, galectin-3 binds *L. major* but this resulted in proteolytic cleavage of galectin-3 and did not promote binding of *L. major* to macrophages. As the N-terminal domain of galectin-3 regulates multimerization and the C-terminal domain of galectin-3 contains the CRD (15), cleaved galectin-3 would be unable to multimerize; thus, galectin-3 could not tether the parasite to host cells, because the bridging effect relies on the multivalency of galectins.

Intriguingly, galectin-mediated bridging of *L. major* to host cells is also important at another point in the parasite life cycle. Valenzuela and co-workers found that a galectin homolog in the midgut of the sand fly *Phlebotomus papatasi* participates in binding of *L. major* at the procyclic phase to gut epithelial cells during infection of this obligate insect host (35). The *L. major* parasite replicates in the sandfly midgut and differentiates into the metacyclic phase that is highly infectious to mammalian hosts and is transmitted during insect bites. Differentiation into the metacyclic phase coincides with alterations in the parasite lipophosphoglycans that reduce binding to the sandfly galectin and allow release of the parasite from the insect gut epithelial cells. Thus, changes in the parasite surface glycoconjugates can promote or reduce binding to various types of galectins in both insect and human hosts at different points during the *L. major* life cycle.

Galectin-3 has also been implicated in enhancing infection of human host cells by the parasite *Trypanosoma cruzi*. Galectin-3 bound to the surface of *T. cruzi* trypomastigotes and exogenous galectin-3 enhanced binding of the parasite to human coronary artery smooth muscle cells. In addition, reducing expression of endogenous galectin-3 by these cells dramatically reduced *T. cruzi* adhesion (36). Galectin-3 also promoted binding of *T. cruzi* to extracellular matrix proteins such as laminin (37). As *T. cruzi* infection increases expression of ECM components such as laminin by host cells, the parasite may use the bridging function of galectin-3 to accumulate in the basement membranes surrounding host target cells such as cardiac myoblasts, thus increasing the likelihood of infection (38).

Most work on the roles of galectins in promoting microbial infection has focused on galectins-1, -3, and -9, that are highly expressed by cells of the immune system as well as endothelial cells and many types of epithelial cells. Other galectins have a more restricted expression pattern; for example, galectin-7 is expressed by squamous epithelial cells. Okumura et al. investigated attachment factors that would promote infection of cervical epithelial cells by *Trichomonas vaginalis* (39). This group found that galectin-1, but not galectin-7, bound to *T. vaginalis*, although both galectins were expressed by cervical epithelial cells. Addition of exogenous galectin-1, but not galectin-7, also enhanced parasite binding to cervical epithelial cells, while reducing expression of endogenous galectin-1 in these cells via siRNA decreased parasite binding. Thus, as has been found for several of the pathogens described above, there is specific binding of one or more distinct galectin family members to different microbial pathogens, demonstrating the unique functions of different galectins at discrete points during infection.

## Galectins Block Microbial Infection

While it is clear that several galectins can bind to microbial glycans to bridge the microbes to host target cells, galectin binding to microbial glycans does not always result in a pro-microbe effect; a microbial glycoprotein that may be available for host attachment may, at another point in the microbial lifecycle, be a liability when galectin binding prevents microbial dissemination. An example of this dichotomy is the interaction of galectin-1 with the NiV–F fusion protein. As described above, binding of galectin-1 to Nipah virus envelope glycoproteins can enhance infection of target endothelial cells by bridging the virus to host plasma membrane glycoproteins (Garner et al., in preparation). However, post-infection, the fusion-promoting activity of the NiV–F protein has a pathogenic effect beyond facilitating viral entry. Endothelial cells infected with Nipah virus and thus expressing NiV–F at the plasma membrane fuse with one another to form giant syncytia; *in vivo*, this process leads to endothelial cell disruption and death and is a primary cause of the hemorrhagic diathesis seen in Nipah virus infection (40–42).

Galectin-1 binding to NiV–F expressed at the plasma membrane prevented cell syncytia formation by three distinct mechanisms. First, NiV–F initially appears at the plasma membrane as an immature precursor that must be endocytosed and proteolytically cleaved intracellularly; the cleaved, mature form recycles to the plasma membrane and is fusion-competent. However, galectin-1 binding to the immature NiV–F protein retards this essential endocytosis step and thus results in decreased production of fusion-competent NiV–F. Second, optimal membrane fusion requires lateral movement of NiV–F on the plasma membrane; however, galectin-1 binding to NiV–F reduces this lateral movement and thus reduces the extent of syncytia formation. Third, NiV–F must undergo a conformational change to effect the membrane mixing required for fusion, and galectin-1 binding inhibits this conformational change of NiV**–**F. While the NiV**–**F glycoprotein has five *N*-glycan attachment sites, one particular glycan, the F3 glycan, on NiV**–**F contributed significantly to all three of these inhibitory effects, i.e., the inhibitory effects of galectin-1 on NiV**–**F maturation, movement, and conformational changes were reduced when the F3 site was mutagenized to remove the *N*-glycan (40, 42). As the NiV**–**F3 glycan was also important for the bridging effect that enhances NiV entry into endothelial cells (Garner et al., in preparation), this demonstrates that the same galectin–glycan interaction that can promote viral entry at an early time point during infection can reduce the pathophysiological effect of viral infection at a later time point.

Finally, the presence of galectin-1 during viral budding reduced the production of Nipah virus particles from infected endothelial cells (42). While it is not yet clear whether this effect resulted specifically and directly from binding of galectin-1 to viral envelope glycoproteins NiV**–**F or NiV–G, or whether binding of host plasma membrane glycoproteins by galectin-1 disrupts efficient viral budding, this observation reinforces the point that the same lectin, galectin-1, can have a pro-viral or anti-viral effect, depending on the timing and context of galectin binding to viral glycoproteins.

Another example of a galectin directly blocking microbial infection is described in the study by Shiau and colleagues (43); this group found that galectin-1 can bind directly to envelope glycoproteins of influenza virus, an effect that impaired virus infection of target cells *in vitro*. In addition to demonstrating this protective effect of galectin-1 *in vitro*, the authors described increased expression of galectin-1 in airway epithelia *in vivo* in a murine model of influenza virus infection. Moreover, intranasal administration of recombinant galectin-1 during influenza virus infection reduced viral load and accompanying inflammation, tissue damage, and mortality in the murine model, and galectin-1 null mice were more susceptible to influenza infection than wildtype mice.

While not directly blocking viral entry, the Panjwani and Argüeso labs found that galectin-3 can bind to ocular mucins to contribute to the barrier function of ocular mucins in preventing infection. Thus, while galectin-3 can bind to HSV-1 to promote viral entry into epithelial cells, as described above (30), galectin-3 also contributes to reducing HSV-1 entry by organizing cell surface mucins to maintain mucosal barrier function in the eye (30, 44), providing another example of a galectin having both infection-promoting and infection-reducing effects for a particular microbial pathogen.

Numerous studies have implicated galectins in promoting a protective innate or adaptive immune response to pathogens, although many of the studies have described effects that do not directly involve binding of the pathogen to a galectin. However, Schwarz and colleagues have demonstrated that the glycophosphatidylinositol (GPIs) on the surface of *Toxoplasma gondii* can bind directly to galectin-3 on the surface of macrophages (45). The binding of these parasite GPIs to cell surface galectin-3, which associates with TLR2, is essential for TLR2-mediated production of tumor necrosis factor (TNF) α by the macrophages. As TNFα is a critical cytokine that promotes parasite clearance, the direct interaction of galectin-3 and *T. gondii* GPIs is an important initial step to reduce the extent of infection.

Finally, several studies have proposed that galectins in the gastrointestinal tract can participate in the organization of mucins into a protective layer that impedes access of microbial pathogens to the host epithelium, implying an indirect role for galectins in reducing microbial invasion. Galectin-4, a galectin that is highly expressed in gut epithelial cells and has been shown to organize epithelial plasma membrane domains by binding to specific glycoproteins and glycolipids, can bind directly to epithelial glycolipids that are receptors for pathogens such as *Bordetella pertussis* and *Helicobacter pylori*. This observation gave rise to the hypothesis that galectin-4 may directly impede the ability of bacterial pathogens to attach to glycolipid receptors and infect host epithelium (46, 47). Experimentally, such a role has been shown for the *Caenorhabditis elegans* galectin LEC8, which has significant homology to mammalian galectins and, like galectin-4, binds glycolipids. Ideo et al. found that LEC8 is expressed in the *C. elegans* digestive tract, and that LEC8 directly blocked infection of the worms with *Escherichia coli*. Moreover, LEC8-deficient animals were more susceptible to *E. coli* infection compared to wildtype worms (48). Thus, depending on the availability of specific microbial glycoprotein receptors for galectins, the anatomic localization of galectin expression, and the timing of galectin expression during infection, galectins may block pathogen binding to and entry into target cells, rather than enhance binding as described in the previous section.

## Galectins have Anti-Microbial Activity

Galectins are evolutionarily ancient molecules found in multicellular fungi, nematodes, and insects as well as vertebrates. Thus, in addition to interfering with pathogen attachment or entry into host cells, as described above, galectins can act as “danger receptors” by either enhancing cytokine production or phagocytic clearance of pathogens by cells of the innate immune system, or by having direct microbicidal activity.

Early work described galectins associating with microbial pathogens in phagocytic vesicles, such as the association of galectin-3 with *Mycobacterium tuberculosis* in macrophage vesicles (49). While a direct anti-microbial mechanism was not determined in this study, galectin-3 null mice had a reduced capacity to clear *M. tuberculosis* late in infection, suggesting that galectin-3 had anti-microbial activity.

A more specific role for galectins in phagocytic clearance of pathogens was recently described for galectin-8 in defense against *Salmonella typhimurium*. *Salmonella* bacteria invade epithelial cells and initially reside in a specialized vacuole. The bacteria generate pores in these vacuoles, damaging the integrity of the vesicle membrane, which allows the bacteria to access the cytosol but also releases “danger signals” that initiate autophagy by the cells to contain the spread of infection. Galectins-1, -3, -8, and -9 are all recruited to vesicles damaged by endocytosed bacteria, and have all been proposed to participate as “danger-sensing” molecules; however, specific actions of galectin-1, -3, and -9 in controlling bacterial damage have not been elucidated. However, Randow and colleagues found that cytosolic galectin-8 is a specific and critical component for activation of autophagy in cells infected with *S. typhimurium* (50). This group has proposed that cytosolic galectin-8 is recruited to the sites of damaged vacuoles by binding to glycan ligands on glycoproteins displayed on the interior face of the vacuoles, although this remains to be definitively demonstrated. Cells lacking galectin-8 failed to recruit other molecular components to the damaged vesicles to initiate autophagy, demonstrating a role for galectin-8 in initiating this critical anti-microbial defense. Randow and colleagues also found that intracellular galectin-8 was recruited to damaged endosomes and lysosomes during infection with *Listeria monocytogenes* or *Shigella flexneri*, as well as during sterile vesicular damage, indicating that galectin-8 is a general component in the autophagy-initiating machinery.

As mentioned above, other galectins have been found associated with endocytosed or phagocytosed pathogens. A direct role for galectin-3 in the phagocytosis of helminths such as *Schistosoma mansoni* has been proposed (51). Galectin-3 can bind GalNAc–β1,4-GlcNAc (LacdiNAc) sequences found on the surface of helminths, and can also bind LacdiNAc ligands that are components of *S. mansoni* soluble egg antigen. Addition of recombinant galectin-3 enhanced recognition and phagocytosis of LacdiNAc-coated beads by rat macrophages, and galectin-3 was abundantly expressed *in vivo* in granulomata containing *Schistosoma* eggs in infected hamsters. Thus, galectin-3 has been proposed to play an important role in the uptake and immunologic “containment” of parasites within granulomata.

A role for galectin-3 in phagocytosis of yeast has also been shown. The yeasts *Candida albicans* and *Candida parapsilosis* can be phagocytosed by macrophages and neutrophils. Bliss and colleagues found that galectin-3 secreted by neutrophils was important for effective phagocytosis of yeast by neutrophils; adding blocking antibody to galectin-3 reduced phagocytosis, while addition of exogenous galectin-3 increased phagocytosis (52). Thus, in neutrophils, galectin-3 appears to act at an early point in the yeast recognition and engulfment process to enhance uptake of fungal pathogens. In contrast, an indirect role for galectin-3 in anti-fungal immunity has also been proposed, as galectin-3 has been found to be important for production of the cytokine TNF-α by macrophages exposed to *C. albicans*. Using a murine macrophage cell line, Fink and colleagues found that galectin-3 was not essential for fungal uptake, but that galectin-3 associated with Dectin-1, a C-type lectin that specifically recognizes fungal β1,3-glucan in the fungal wall and directly mediated fungal uptake; loss of galectin-3 expression in these cells did not reduce fungal uptake, but reduced TNF-α production (53). Similarly, Poulain and colleagues found that, while galectin-3 can bind specific β1,2-mannosides on *C. albicans* mannoproteins, *C. albicans* uptake by human monocyte-derived macrophages required TLR2 but not galectin-3; however, galectin-3 associated with TLR2 in macrophages that had phagocytosed yeast, and galectin-3 expression by the macrophages was essential for optimal TNF-α production after yeast phagocytosis (54).

In addition to promoting phagocytic uptake or autophagic destruction of bacteria, fungi, and parasites, galectins have been shown to have direct microbicidal activity. As mentioned above, galectin-3 can recognize specific fungal mannosides on *C. albicans*. In examining the binding of galectin-3 to intact, live *C. albicans*, Kohatsu et al. observed that galectin-3 binding was directly fungicidal (55). The effect was dependent on galectin-3 binding to specific glycan ligands on the yeast, and the fungicidal effect was only seen with *Candida* species that expressed specific β1,2-oligomannans. Galectin-3 binding to susceptible yeast resulted in morphologic changes, including cell shrinkage and increased intracellular granularity. Cell death was confirmed by a number of assays, and direct fungicidal, rather than fungistatic, activity was confirmed. While the mechanism of fungal death has not been precisely determined, galectin-3 binding appeared to directly damage the integrity of the cell membrane, given the observed cell shrinkage and uptake of a fluorescent dye that would be excluded by intact cells.

A direct microbicidal effect was also demonstrated for galectin-4 and -8 in the killing of *E. coli* that express the blood group B glycans on the cell surface (56). Unlike the autophagy-promoting activity of intracellular galectin-8 described above (50), addition of either galectin-4 or -8 to susceptible *E. coli* strains in the absence of any host cells resulted in rapid killing of the bacteria, as demonstrated by loss of membrane integrity and failure of the bacteria to divide. The complement-independent, rapid, and direct damage to the bacterial membrane is reminiscent of the activity of galectin-3 on susceptible *Candida* species, described above. As with *Candida*, the mechanism of bacterial death triggered by galectin binding has not been determined; however, it is tempting to speculate that the cross-linking of numerous glycoprotein or glycolipids on the pathogen surface by galectin binding results in physical changes or mechanical stresses to the surface or interior of the pathogen that compromise cellular integrity. Stowell and colleagues propose that the ability of galectins-4 and -8 to directly kill bacteria is part of an innate immune function of galectins, providing an important line of defense against gastrointestinal pathogens.

While not a mammalian defense against microbial pathogens, another form of microbicidal activity by galectins has been described for the conger eel (57). This organism expresses two galectin family members, congerin I and II, in the digestive tract. In eels infected with parasitic *Cucullanus* nematodes, these galectins mediate adhesion of nematodes to peritoneal cells that encapsulate and sequester the parasites. This effect may be similar to the effect of mammalian galectin-3 sequestering *Schistosoma* eggs in granulomata in mammalian hosts; while not directly microbicidal, the effect of encapsulation or sequestering of microbial pathogens prevents pathogen replication and protects the host.

## Conclusion

Galectins have been described as PRRs that can discriminate microbial glycans from host glycans, and as danger receptors that can participate in innate immune defense. These are certainly functions of the galectins, but it is becoming increasingly clear that galectins can bind both host and microbial glycans; thus, assuming that there is a non-overlapping dichotomy of pathogen vs. host glycans is not valid. Rather, galectins bind a variety of glycans in a context-dependent manner on both microbes and on mammalian cells (58). Moreover, the outcome of binding can be quite variable, both promoting infection and blocking infection, and both directly killing microbes and initiating immune responses; as pathogens evolve in this environment of host glycans and host galectins, microbes may adapt by exploiting these host features to enhance infection or evade an immune response (59–62). This broad array of functional outcomes does not reflect a lack of specificity of the galectins, but rather emphasizes that specific responses are dependent on both the pathogen and the host cell and are determined by factors such as glycan density, glycan clustering, glycan presentation on specific glycoproteins and glycolipids, and interactions with other cell surface molecules, all of which are features that define unique cellular landscapes in which the galectins act. Thus, as more examples of galectin–microbe interactions are discovered, it may be possible to begin to define features of the galectins and glycans that would predict the outcome of a particular binding event. As galectins are evolutionarily ancient molecules, predating vertebrate innate and adaptive immunity, it is not surprising that these lectins have evolved into a large family with a wide range of context-specific functions.

## Conflict of Interest Statement

The authors declare that the research was conducted in the absence of any commercial or financial relationships that could be construed as a potential conflict of interest.
